# Manipulating Bacterial Biofilms Using Materiobiology and Synthetic Biology Approaches

**DOI:** 10.3389/fmicb.2022.844997

**Published:** 2022-07-07

**Authors:** Yue Shi, Tingli Chen, Peter Shaw, Peng-Yuan Wang

**Affiliations:** ^1^Oujiang Laboratory, Key Laboratory of Alzheimer’s Disease of Zhejiang Province, Institute of Aging, Wenzhou Medical University, Wenzhou, China; ^2^Shenzhen Key Laboratory of Biomimetic Materials and Cellular Immunomodulation, Shenzhen Institute of Advanced Technology, Chinese Academy of Sciences, Shenzhen, China; ^3^College of Life Science, University of Chinese Academy of Sciences, Beijing, China

**Keywords:** biofilm, bacteria, materiobiology, extracellular matrix, gene editing

## Abstract

Bacteria form biofilms on material surfaces within hours. Biofilms are often considered problematic substances in the fields such as biomedical devices and the food industry; however, they are beneficial in other fields such as fermentation, water remediation, and civil engineering. Biofilm properties depend on their genome and the extracellular environment, including pH, shear stress, and matrices topography, stiffness, wettability, and charges during biofilm formation. These surface properties have feedback effects on biofilm formation at different stages. Due to emerging technology such as synthetic biology and genome editing, many studies have focused on functionalizing biofilm for specific applications. Nevertheless, few studies combine these two approaches to produce or modify biofilms. This review summarizes up-to-date materials science and synthetic biology approaches to controlling biofilms. The review proposed a potential research direction in the future that can gain better control of bacteria and biofilms.

## Introduction

Bacteria exist ubiquitously in nature and can adhere to nearly any type of surface, including biotic, abiotic, and natural surfaces. In many cases, it is beneficial for bacteria to transform from a single-cell planktonic lifestyle to a multicellular assembled community mode—biofilm. The biofilm is not only simply clusters of bacteria but also involves essential physiology and phenotypic changes. It is a highly structured multicellular microbial community composed of bacteria cells and an extracellular polymeric substance (EPS) matrix (Verderosa et al., 2019; Arnaouteli et al., 2021). Biofilms can be found on the surface of river rocks, seaside reefs, the roots of plants, deep-sea animal epidermis, water pipelines, food processing equipment, and even medical facilities. The problems caused by biofilms include economic loss and health-related issues since bacteria in a biofilm can be protected from the harsh environment, including the mechanical shear stress of fluid flow, antibiotics, or chemical interference (Schultz et al., 2011; Hall and Mah, 2017; Del Pozo, 2018; Cheng et al., 2019; Abebe, 2020). It is reported that the annual cost for cleaning and coating surfaces of navy ships alone in America is about US$56 million due to damage caused by matured biofilms (Hall and Mah, 2017). In the food-processing field, biofilms form on the processing line is a severe public health concern due to the potential causes of food contamination and foodborne diseases (Abebe, 2020). In addition, infections associated with biofilms developed on medical devices are also a persistent challenge for health care since the antibiotic resistance of biofilm may increase approx. 100- to 1,000-fold compared to their planktonic state (Hall and Mah, 2017; Del Pozo, 2018). The requirements for preventing biofilm formation or removing matured biofilm have been a long-standing focus of research efforts. Techniques used for antimicrobial purposes, including surface modifications and drug developments, have been well documented in some reviews (Rigo et al., 2018; Kyzioł et al., 2020; Valenzuela et al., 2021).

However, biofilms do not always cause negative implications. They can become a powerful tool in industries or agriculture by taking advantage of their “intelligent” characteristics such as systematic growth, self-repairing, and response to environmental signals to switch their growing state (Todhanakasem, 2017; Romero et al., 2018; Zhao et al., 2019; Pandit et al., 2020; Singh and Kaushik, 2021). For example, a biofilm’s ability to biotransformation can be used for wastewater treatment in bioremediation (Zhao et al., 2019; Singh and Kaushik, 2021). It has been demonstrated that biofilm’s high-cell density, immobilization, and stability could be ideal features. Moreover, since biofilms have little sensitivity to medical drugs and toxins and are highly active organized reaction systems, they can act as a biocatalyst in fabricating many bio-based products, such as antibiotics, enzymes, exopolysaccharides, bioenergy, and biorefinery (Todhanakasem, 2017; Romero et al., 2018).

**FIGURE 1:**
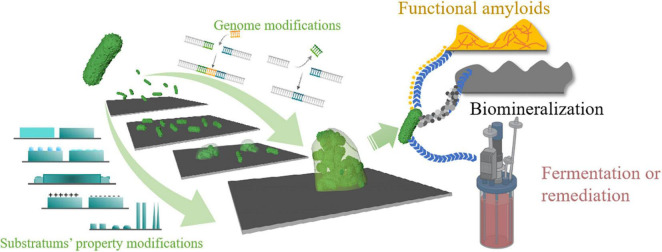
Schematic illustration of the review. Bacteria can form biofilms once attached to a substrate that can be beneficial or problematic depending on specific situations. A thorough understanding of biofilm formation factors and their properties is necessary for better application. Both genomic modifications of the bacteria and substratum’s properties can affect biofilm’s properties. Currently, little research has focused on combining these two methods to control biofilm formation. In this article, we propose that by taking advantage of both synthetic biology and materiobiology, it is possible to gain better control over biofilm formation.

To control biofilm formation, a thorough knowledge of factors influencing the biofilm formation process is crucial for both prevention and application. In addition to its genome, biofilm formation can also be affected by environmental factors, including flow rate, environmental pH, temperature, gravitational force, and substratum surface properties (Linklater et al., 2018; Ren et al., 2018; Achinas et al., 2019; Secchi et al., 2020; Krsmanovic et al., 2021; Nakanishi et al., 2021). In this review, we neither treat biofilms as threats nor beneficial but focus on controlling biofilm formation from synthetic biology and substratum surface properties. Both of these aspects could influence biofilm formation to some extent. We propose that combining these two techniques might better control the biofilm and thus benefit a range of applications (Figure 1). We have structured this review into two parts: the first part will discuss recent updates on the effects of substratum surfaces’ properties on biofilm formation. In contrast, the second part will give representative applications using synthetic biology methods to control biofilm formation.

## Controlling Biofilm Formation Using Materials Science

It is widely accepted that the development of biofilms includes the following four stages: (1) initial attachment; (2) microcolony formation; (3) biofilm maturation; and (4) biofilm dispersal (Zhao et al., 2017; Arciola et al., 2018). Bacterial adhesion is the first step for biofilm formation on the surface. Both reversible and irreversible attachment appear at this stage where only the latter can yield those attached bacterial continue to grow into microcolonies. Microcolonies, the basic units in most biofilms, start to form when cells proliferate on surfaces. The biological processes of the bacteria dominate this period and are characterized by the excretion of EPSs and signaling molecules. EPS, consisting of various extracellular polysaccharides, DNA, and proteins, is critical for bacteria to construct and maintain the biofilm structure. Still, the composites and properties of EPS vary significantly by species and environment (Zhao et al., 2017; Arciola et al., 2018). The biofilm then starts to mature with the microcolonies growing and expanding to reach a steady state where nutrient transportation and cellular activities are balanced. The fluid-phase channels contain either nutrients or wastes interspersed within the mature biofilm, creating pillars segregating different microenvironments that translate to various cell activities, making it a complex, highly differentiated cell community (Zhao et al., 2017; Arciola et al., 2018). The last stage of the biofilm development is dispersal, in which cell departs from biofilm and converts to the planktonic mode of growth (Zhao et al., 2017; Arciola et al., 2018). In addition to the bacteria’s genome and environmental factors, the substratum’s surface properties also play an important role during biofilm formation. These surface properties include topography, roughness, stiffness, surface charge, and hydrophobicity. Here, we summarized some representative studies on how substratum properties affect biofilm formation.

### Stage 1—Bacteria Adhesion

#### Topography and Roughness

Specific patterns and roughness changes can characterize surface topography changes. By specific patterns, we refer to the topography containing certain shapes and the pattern size, usually at micro and sub-micro scale-like pillars, grooves, wire, etc. Roughness changes are irregular shapes at the nanoscale level (Tables 1, 2).

**TABLE 1:** Summary of the effects of patterned substrates on bacterial adhesion stage.

Material	Pattern	Parameters	Strains	Time	Conclusion	References
PDMS	Square Circular Ridges	D: 3 μm H: 21.1, 117 nm S: 2, 4 μm R: 9.6–55 nm	*Staphylococcus epidermidis* and *Staphylococcus aureus*	30 min, 8 h	Topography reduced bacterial adhesion (40–95%) and biofilm formation (22–58%)	Vadillo-Rodriguez et al., 2018
SU-8 photoresist glass slides	Micropillar arrays	D: 5,13 μm H: 5 μm S: 10, 20 μm	*Escherichia coli*	168 h	Pillar topography did not reduce the coverage of *E. coli*	Encinas et al., 2020
PDMS	Line patterns	D:5, 10, 20 μm H: 5 μm S: 3, 5, 10, 20 μm	*Escherichia coli*	24 h	*E. coli* cells prefer to align perpendicularly to the direction of narrow line patterns	Gu et al., 2016a
Titanium alloy	Nanopillars	D: 620–880 nm H: 135–215 nm R: 0.1–0.5 μm	*Staphylococcus aureus*	48 h	Nanopillars inhibited bacterial colonization and bacteria retention	Cunha et al., 2016
Stainless steel	Cones holes	D: 55, 68 nm	*Escherichia coli* and *Staphylococcus aureus*	2 h	Cones and holes yielded significant reductions of *E. coli* and *S. aureus*	Peter et al., 2020
glass Slides silicone	Filaments rods		*Escherichia coli* and *Staphylococcus epidermidis*	3 h	Topography reduced the number of adherent bacteria in static condition	Meier et al., 2018
APTES, SU8 TAF	Hole, post, line	Λ = 500, 1,000, 5,000 nm	*Escherichia coli* and *Staphylococcus epidermidis*	2 min, 24 h	A larger size leads to high cell retention	Helbig et al., 2016

*D, diameter; H, height; S, space; R, roughness; Λ, periodicities.*

**TABLE 2:** Summary of the effects of substrates’ roughness on bacterial adhesion stage.

Material	Pattern	Roughness	Bacteria	Time	Conclusions	References
Ti EG2/4	Irregular nanoarchitecture	0.16–0.86 nm	*Staphylococcus aureus* and *Pseudomonas aeruginosa*	18 h	Ra < 1 nm could inhibit the *P. aeruginosa* colonization but no difference could be found in *S. aureus* attachment patterns	Truong et al., 2015
Ceramic	Irregular nanoarchitecture	1.5–205 nm	*Staphylococcus aureus*	24 h	The number of bacterial adhesions on the surface of Ra 1 nm is much less than that of Ra 205 nm	Lu et al., 2020
Pure ASTM Grade-2 titanium	Irregular nanoarchitecture	3.80 ± 1.39 nm	*Staphylococcus aureus* and *Pseudomonas aeruginosa*	18 h	More number of *S. aureus* and *P. aeruginosa* attach to surfaces with smaller roughness	Truong et al., 2010
Gold-coated wrinkled polystyrene surfaces	Irregular nanoscale wrinkles	41, 258 nm	*Pseudomonas aeruginosa* and *Staphylococcus aureus*	18 h	Reduced *P. aeruginosa* attachment to 57% and *S. aureus* attachment to 20%, respectively	Nguyen et al., 2018
Stainless Steel	Irregular microarchitecture	45.2–172.5 nm	*Pseudomonas aeruginosa* and *Staphylococcus aureus*	24 h	Much smaller number of *P. aeruginosa* and *S. aureus* cells attached to the electropolished surfaces	Wu et al., 2018a
Ti, PET	Irregular microarchitecture	4–170 nm	*Staphylococcus aureus*	24 h	A significantly decreased bacterial adhesion for structures with an aspect ratio range of 0.02 to 0.05	Meinshausen et al., 2021

Surface patterns can alter bacterial adhesion behaviors (Table 1). Specific-sized patterns like nanoneedles or pillars can affect bacterial viability (Wu et al., 2018b; Ivanova et al., 2020; Jiang et al., 2020; Xiao et al., 2020). These antimicrobial patterns have been well summarized in other literature, which is not our focus (Linklater et al., 2020; Mahanta et al., 2021). In this discussion, we focus on the bacteria behaviors when adhering to “mild patterns,” i.e., the patterns are not fatal to the majority of attached bacteria (Table 1). Gu et al. studied *Escherichia coli’s* adhesion behaviors on gratings. They found that the orientation of attached bacteria was more perpendicular to the orientation of gratings when the widths decreased. The attached cell shape was longer and their transcription activity was higher on the narrow gratings (Gu et al., 2016a). Silica and polystyrene particle’s self-assembled surfaces showed that *E. coli* and *Staphylococcus aureus* preferred attaching to the valley area instead of growing across the particle (Shi et al., 2021). In addition to attachment locations, patterns also affect the number of bacteria attached. Pattern size and spacing were critical because smaller-sized pillar patterns showed low fouling effects for *S. aureus* and bactericidal effects for *E. coli.* In contrast, larger-sized patterns showed low fouling effects for *E. coli* and patterning toward *S. aureus* (Heckmann and Schiffman, 2020). By fabricating either protruding or recessing grooves, square and circular features on PDMS surfaces *via* soft lithography with various parameters, it was demonstrated that patterned surfaces not only reduced *Staphylococcus epidermidis* and *S. aureus* attachment but can also alter the bacteria attachment locations. Bacteria actively choose their settling position based on the cell-surface contact points maximization principle (Vadillo-Rodriguez et al., 2018). The authors ruled out the possibility that the low-fouling effects were due to physical constraints or surface hydrophobicity. They proposed that it may be caused due to the nanoscale surface roughness-induced interaction energies (Vadillo-Rodriguez et al., 2018). Other than highly ordered patterns, random surface features, such as filaments and irregular protruding features, can also reduce the attachment of bacteria (Cao et al., 2018; Meier et al., 2018; Peter et al., 2020), but not all patterns have a passive effect on bacterial adhesion. Encinas et al. (2020) showed that there were more bacteria colonies adhered on micropillars than on flat and nanofilament surfaces, and explained that this association is due to the size of micropillars being larger than bacteria, which gives sufficient space for bacteria to anchor and adhere.

Studies have shown that even on the nanoscale, surfaces still can affect bacterial adhesion. Surface roughness was usually impacted by material processing steps such as direct laser interference patterning (DLIP) (Meinshausen et al., 2021), plasma etches (Linklater et al., 2019), and Femtosecond laser (Table 2; Cunha et al., 2016). The roughness of the surface varied in nanoscales, which was much less than the size of bacteria. Wu et al. found that the membrane of bacterial cells on the surface with nanoscale topography would be slightly deformed and elongated. They suggested the nanoscale surface topography could inhibit the bacteria’s adhesion and proliferation (Wu et al., 2018a).

Nevertheless, it is difficult to conclude whether a smooth surface reduces or increases bacterial attachment. Jang et al.’s prepared nanostructured stainless steel 316L by electrochemical etching. They found fewer bacteria were attached to the nanostructured surfaces than the smoother control ones (Jang et al., 2018). Later, in 2020, Bilgili et al. (2020) claimed no statistically significant differences were observed among different bulk-fill composite resins in terms of roughness. However, in 2021, Aouame et al. reported that the bacteria adhesion and biofilm formation mainly depend on surface characteristics. Bacteria are more likely to adhere to and develop biofilm on rough dental surfaces than on smooth stainless-steel surfaces (Aouame et al., 2021). Different materials, strains, and test methods were applied in these studies, making it difficult to compare them.

#### Stiffness

**FIGURE 2:**
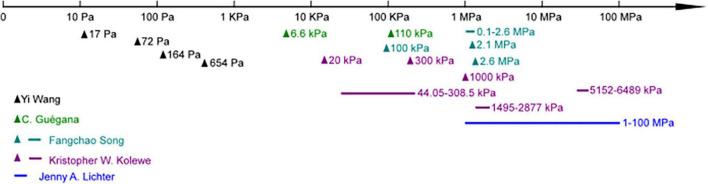
Studies focused on different substrate moduli ranges in bacteria adhesion.

It is well known that substrates’ stiffness plays a vital role in mammalian cell attachment, migration, differentiation, and tissue homeostasis process (Handorf et al., 2015; Spencer et al., 2017). Fewer studies cover this area regarding their effects on the behavior of bacteria, and the results are still controversial (Figure 2 and Table 3). The discussed substrates here were chemically and mechanically stable.

**TABLE 3:** Summary of the effects of substrates’ stiffness on biofilm adhesion stage.

Material	Young’s moduli	Strain	Findings	References
PDMS	0.1–2.6 MPa	*Escherichia coli*	More E. coli cells attached to the surface of softer PDMS	Song et al., 2017
PEGDMA	44.05–308.5 kPa; 1,495–2,877 kPa; 5,152–6,489 kPa	*Escherichia coli* and *Staphylococcus aureus*	The number of cells increases with the stiffness of the hydrogel	Kolewe et al., 2015
EG	20 kPa; 300 kPa; 1,000 kPa	*Escherichia coli* and *Staphylococcus aureus*	Bacteria attachment increased with increasing hydrogel stiffness	Kolewe et al., 2018
Agarose	6.6 kPa; 110 kPa	*Pseudoal-teromonas* sp., *Bacillus* sp.	More Ps. adhere to the stiffer surface, Bs. form clusters on the softer surface	Guegan et al., 2014
PDMS	0.26 kPa; 124 kPa	*Escherichia coli*	Bacteria attached more strongly to soft surfaces compared to stiff ones	Siddiqui et al., 2019
PDMS	2.6 MPa; 1.0 MPa; 0.1 MPa	*Escherichia coli* and *Pseudomonas aeruginosa*	More cells are attached to the softer surfaces. The cell size on the stiffer surface is smaller	Song and Ren, 2014
PAAm	654 Pa; 164 Pa; 1.72 Pa; 17 Pa	*Staphylococcus aureus*	More bacteria cells adhered to the stiffer surface	Wang et al., 2016
PEM	1–100 Mpa	*Staphylococcus epidermidis*	More cells adhered to the stiffer surface	Lichter et al., 2008

Lichter et al. were the first to report that substrate stiffness could affect the adhesion of bacteria independent of other physicochemical properties, including roughness, interaction energy, surface charge density, and monovalent ion concentration. By assembling polyelectrolyte multilayers on Ti substrates under different conditions, the stiffness of such hydrated films could be varied between 1 and 100 MPa. Their studies applied the Gram-positive strain *S. epidermidis* and Gram-negative strain *E. coli* (wild-type and *mreB* mutant strains). They concluded that the adhesion of bacteria correlates positively with increasing elastic modulus over the testing range (Linklater et al., 2019). Later, Rachel’s group proved Gram-negative bacterial strain *Pseudoalteromonas* sp. D41 had similar trends by using agarose hydrogel as a substrate. Besides, they identified 21 proteins differentially regulated when attached to different stiffness *via* a proteomic approach. Most of these proteins are involved in key metabolic pathways, suggesting the substrate’s stiffness affects both the bacterial adhesion amount and their phenotype. Furthermore, concerning Gram-positive *Bacillus* sp. 4J6, though the adhesion pattern changed with stiffness, the number of attached bacteria showed no statistical difference (Guegan et al., 2014). Similarly, by using poly-(ethylene glycol) (PEG) hydrogel, Schiffman’s group showed the amount of bacterial attachment (*E. coli* and *S. aureus*) was positively correlated with hydrogel stiffness (Kolewe et al., 2015, 2018). Song and Ren (2014) and Song et al. (2017), Moraes (Siddiqui et al., 2019), and Phillips (Wang et al., 2016) research groups drew conflicting findings on how bacteria attached to soft substrates. Phillips’s group used polyacrylamide (PAAm) hydrogel with elastic modulus ranging from 17 to 654 Pa, and found that the adhesion rate and numbers of adherent *S. aureus* decreased with increased modulus (Wang et al., 2016). The other two groups used PDMS as substrates, and both found a higher number of *E. coli* attached to soft substrates. In addition to the attached amount, Ren’s group found the length of bacteria attached on the soft surface was longer than stiff ones (Song and Ren, 2014). Moraes’s group studied the effects of shear force and bioactive molecules coating in addition to substrate stiffness. With ECM molecules coating, bacterial adhesion strength generally decreased, but these effects were smaller than those caused by substrate mechanical properties. They demonstrated that bacteria attached evenly on soft and hard surfaces under low shear force, but a higher number of bacteria retained on soft surfaces while exposed to higher shear. They claimed that the sheer force might be one of the reasons causing contradictory results in the literature (Siddiqui et al., 2019).

The studies covering the underlying mechanism of surface stiffness affecting bacteria were even less. By culturing bacteria in different divalent or monovalent ion concentration solutions, the activation of transient receptor potential ion channels was proved not required for *S. epidermidis* sensing of mechanical stimuli. By comparing mutant strain and wild type together with *S. epidermidis*, it was shown that the shape of bacteria was also not the reason (Lichter et al., 2008). The behaviors of *E. coli RP437* and three of its isogenic mutants of *motB, fliC*, and *fimA* genes were compared and confirmed it is the *motB* gene involved in the response of *E. coli* to PDMS stiffness during the attachment stage (Song and Ren, 2014).

#### Other Parameters

Other surface properties such as surface charge and hydrophobicity can also affect the bacterial adhesion stage. Highly positively charged surfaces are usually considered bactericidal since they can rupture the cell membrane through electrostatic attractions (Pranantyo et al., 2018; Salama et al., 2020). When surface charge density is below the critical value of bactericidal, it will influence bacteria adhesion behaviors. Layer by layer assembly of cationic/anionic polymers is one of the coating methods for adjusting materials’ surface charges. It was shown that positively charged surfaces attracted bacteria compared to the negative ones due to the original negative potential of the bacterial cell wall (Zhu et al., 2015; Kovačević et al., 2016; Guo et al., 2018). Besides, there was a positive correlation between the adsorption of bacteria and the surface charge (Chen C. et al., 2019). Similar trends were also found by carefully designed polymer chain coating to control surface hydrophobicity and surface potential (Rzhepishevska et al., 2013; Oh et al., 2018). The phenomenon of *Pseudomonas aeruginosa* oriented vertically on negatively charged surfaces was suggested due to the cell’s intention to minimize the exposure to the repel force between their membrane and the surfaces (Rzhepishevska et al., 2013). However, there are also some controversial conclusions where some studies showed that regardless of the material chosen, the surface charge had no impact on the bacterial attachment (Lorenzetti et al., 2015; Spriano et al., 2017).

Wettability is defined as the ability of a liquid to wet a surface and is usually determined by the contact angle. Though it is generally believed that hydrophobic cells tend to adhere to hydrophobic surfaces while hydrophilic ones to hydrophilic surfaces, the relationship between surface wettability and bacterial adhesion is complicated to discuss on its own because wettability depends on many other surface parameters as well, such as roughness, surface charge, surface chemistry, and so on (Krasowska and Sigler, 2014; Jothi Prakash and Prasanth, 2021).

Conditioning films start to form once substratum surfaces are in contact with aqueous environments where various organic and inorganic compounds are adsorbed onto the surfaces. This conditioning film influenced initial bacterial adhesion by altering substratum surface charge, hydrophobicity, roughness, and chemical composition (Talluri et al., 2020; Bhagwat et al., 2021; Wurzler et al., 2022). Whether this conditioning film promotes or inhibits adhesion highly depends on the status presented on the surfaces (Berne et al., 2018; Wurzler et al., 2022).

It is worth noticing that, when choosing their behaviors, bacteria would seem to comprehensively consider all surface properties. These surface properties might only have one or two factors predominant bacterial behaviors or, sometimes, may all have a contribution (Spriano et al., 2017; Wassmann et al., 2017; Aouame et al., 2021). Besides, the testing conditions, such as culture media used, either flow system or static system, will all affect the bacterial attachment (Senevirathne et al., 2021).

### Stage 2—Microcolony Formation

**FIGURE 3:**
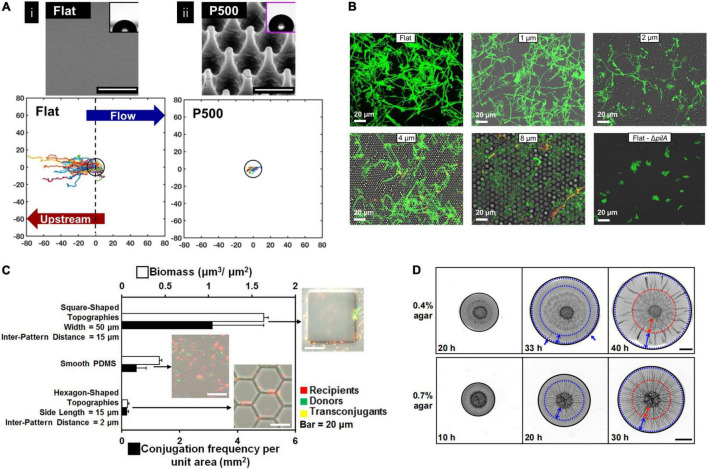
**(A)** Nanopillared topography inhibited bacteria upstream motility indicated by single-cell trajectories. Single-cell trajectories of *Pseudomonas naeruginosa* on the flat surfaces originated from 0 and extended to the –80 μm in *x*-direction indicating upstream motility, while much less motility distance was observed on a nanopillar topography (P500). The black ring is 10 μm radium as a reference (Rosenzweig et al., 2019). Adapted with permission from Rosenzweig et al. (2019). Copyright 2019 American Chemical Society. **(B)** Hemisphere topography affected bacterial migration. Fluorescence images showing *P. aeruginosa* movement traces. It showed bacteria appear to explore a smaller fraction of the flat surface compared with hemisphere topography surfaces with 2–8 μm features (Yow-Ren Chang and Ducker, 2018). Adapted with permission from Yow-Ren Chang and Ducker (2018). Copyright 2018 American Chemical Society. **(C)** Conjugation frequency and biofilm formation of *Escherichia coli* were influenced by surface topography. The square-shaped pattern showed higher conjugation frequency than smooth and hexagon-shaped surfaces (Gu et al., 2017). Adapted with permission from Gu et al. (2017). Copyright 2017 American Chemical Society. **(D)** Representative images of *Vibrio cholerae* biofilm grown on different substrates at different time points. Blue dotted circles mark the boundaries of regions with radical patterns, and red dotted circles mark the boundaries of regions with zigzag patterns. It showed biofilm morphology differed on different substrate’ stiffness (Fei et al., 2020). Adapted with permission from Fei et al. (2020). Copyright 2020 National Academy of Science.

Substrates’ properties can affect bacterial behaviors during biofilm formation. Poly(methyl methacylate) (PMMA) surface with sub-cellular nanopillar topography not only can inhibit *P. aeruginosa* attachment but also prevent upstream movement and reduce the proliferation of bacteria cells (Figure 3A; Rosenzweig et al., 2019). On a micrometer-scale surface with a crystalline hemispherical pattern, bacteria behave differently depending on the diameter of the raised features. A diameter of 2 μm and larger hemispherical patterns could hinder *P. aeruginosa* motility, while a 1 μm pattern showed no significant difference compared with a flat surface. In addition, their travel directions also differed by diameters. On 2 μm surfaces, bacteria were more likely to move in a reverse direction. On 4 μm surfaces, they prefer to travel in approximately straight lines in the groove along the crystal axis. In comparison, they were more likely to follow an approximately hexagonal lattice of grooves on 8 μm surfaces since this feature was complex for bacteria to move across the crowns (Figure 3B; Yow-Ren Chang and Ducker, 2018). On a stepped topography, *P. aeruginosa* drastically reduced the probability of crossing the step compared with crossing a point on a flat surface and significantly reduced the speed perpendicular to the step when they were very close to the step. When step height was similar to the bacterial length, a time penalty to cross the step was found, while no time penalty was resolved to cross tall steps (5–9 μm) (Chang et al., 2019). The critical length-scale for topography affecting *P. aeruginosa* movements seems to be similar to the dimensions of the bacterium and the length of pili. They proposed that if the topography in a particular direction is not favorable for pili attachment, then that direction of motion may also be disfavored (Chang et al., 2019). Another study also reported this threshold of topographical barriers of ∼1 μm using a different pattern. It was found that a 1 μm depth of micro-fabricated furrows can inhibit the expansion of *P. aeruginosa* biofilm more effectively than a 0.5 μm depth one (Gloag et al., 2016). For *E. coli*, a 10 μm tall hexagon-shaped topographic pattern with a side length of 15 μm and inter pattern distance of 2 μm showed the interrupting biofilm formation effects. In contrast to the smooth surfaces, biofilm formation and conjugation were promoted for larger square-shaped patterns with side lengths larger than 20 μm and inter pattern distances no less than 10 μm. Besides, compared to the top and grooves of the pattern, the vertical sidewall was favored for bacterial conjugation (Figure 3C; Gu et al., 2017).

In addition to topography, substrate stiffness also affects the biofilm formation process. It has been found that the average velocity of *E. coli* movement was higher on rigid PDMS surfaces compared to the soft ones (Song et al., 2017). By establishing an idealized bacteria twitching mathematical model, Sabass’ group analyzed how substrate rigidity influenced bacterial migration and revealed that migration depends on the force-sensitivity of the adhesion bond. The results showed that bacteria migration speed depends non-linearly on substrate rigidity. Bacteria move faster on a rigid surface if their rear adhesion bond is more force-sensitive. However, the deformation of the soft substrate surface may block the pull force produced during migration, which negatively affects migration speed (Simsek et al., 2019).

Furthermore, substrate stiffness can also affect the pattern of growing biofilms. It has been reported that the *Vibrio cholerae* biofilm spread varied on the surface of different agar concentration hydrogels. The morphology of growing *V. cholerae* biofilms is fold-shaped, extending from the edge to the center on the soft hydrogel surface of low agar concentration. In contrast, in the high agar concentration hydrogel surface, the folds extend in the opposite direction from the center to out edge. The sliding friction between agar surface and biofilm may contribute to the morphology difference during biofilm expansion (Figure 3D; Fei et al., 2020).

### Stage 3—Biofilm Maturation

During maturation, bacteria often use quorum sensing to coordinate each other and the community. A cell–cell communication system involves the production, release, accumulation, and detection of autoinducers depending on the cell density, species composition of the microbial community, and the surrounding environment. As the population density of bacteria communities increases, the amount of autoinducer accumulates in the surrounding environment, which further affects the global gene expression patterns and cell-to-cell interactions (Papenfort and Bassler, 2016; Abisado et al., 2018). The key to quorum sensing is that bacteria cells receive, recognize, and respond to these signal molecules. Many studies have demonstrated that using molecular biology techniques to alter the bacterial quorum-sensing system will affect bacterial metabolic activities and biofilm formations. Detailed information has been well-reviewed in other articles, which is not our focus here (Mukherjee and Bassler, 2019; Li and Zhao, 2020; Wang N. et al., 2021).

From a material point of view, autoinducer peptide -I (AIP-I) is an essential inducer of the *S. aureus* Agr quorum-sensing system and can be detected by a cognate transmembrane bound receptor. This molecule could be immobilized onto a glass substrate *via* a flexible linker to influence the biofilm formation process (Kim et al., 2017). Similarly, a synthetic quorum sensing inhibitor 5-methylene-1-(prop-2-enoyl)-4-(2-fluorophenyl)-dihydropyrrol-2-one can be covalently incorporated into the surface coating to hindering bacterial communication (Ozcelik et al., 2017).

**FIGURE 4:**
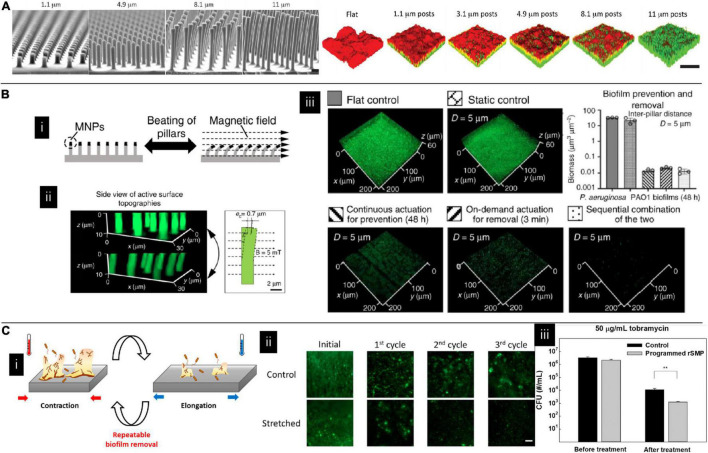
**(A)** Topography affecting multispecies biofilm morphology and species content where green and red colors indicate *Escherichia coli* and *Pseudomonas aeruginosa*, respectively. The fraction of *E. coli* in the co-cultured film increased with the pillar height (Bhattacharjee et al., 2017). Adapted with permission from Bhattacharjee et al. (2017). Copyright 2017 American Chemical Society. **(B)** The effect of magnetically driven dynamic pillar pattern on biofilm formation. **(i)** Schematic image of pillar bending in response to an external magnetic field. **(ii)** Fluorescent images of the pillar before and after exposure to a magnetic field. **(iii)** Representative fluorescence images of biofilms on flat controls, static controls, and active surface topographies (Gu et al., 2020). Adapted with permission from Gu et al. (2020). Copyright 2020 Spring Nature. **(C)** The effect of shape recovery substrate on biofilm formation. **(i)** Schematic illustration of dynamic substrates. **(ii)** Biofilm staining on different dynamic substrates. **(iii)** Detached bacteria were more susceptible to antibiotics on dynamic substrates (Lee et al., 2021).

The substrates’ properties can still have an impact on the matured biofilms. For example, it has been found that micropatterned pillar surfaces could modulate competition dynamics and signaling pathways in the co-culture environment of *E. coli* and *P. aeruginosa.* With the increased height of the pillar, the biofilm featured increasing volume fractions of *E. coli* cells. They explained that this phenomenon resulted from accumulating a signaling molecule indole, which can silence *E. coli’s* biofilm dispersal and inhibit *P. aeruginosa’s* formation. Besides, the authors also found that micropatterned pillar surfaces could affect their antibiotic susceptibilities in mono-species culture. Increased susceptibility was found in patterns with the higher pillar (Figure 4A; Bhattacharjee et al., 2017). In addition to topography, the surface charge can influence biofilm structure. For example, *P. aeruginosa* biofilms form mushroom shapes on negatively charged surfaces while flat on positively charged surfaces. This phenomenon is due to the negatively charged surface inhibiting motility, preventing bacteria from moving over the surface to create a flat biofilm (Rzhepishevska et al., 2013). Besides, substrates’ stiffness also affects biofilm’s susceptibility; biofilms growing on stiff substrates were less susceptible to antibiotics than those on the soft substrates (Song and Ren, 2014).

In addition to influencing naturally formed biofilms, materials could also assist bacteria in forming biofilms for better performance (Hubenova et al., 2019; Reinhardt et al., 2020). For example, alginate could be functionalized with thiazolyl blue formazan/phenazine methosulfate and showed good biocompatibility of immobilization of bacteria *Pseudomonas putida* 1046 for constructing artificial biofilms. This artificial biofilm exhibited good electrochemical activities due to the incorporated redox-active cross-linking network in the surrounding polymer matrix around the microbial cells. They were considered good candidates for bioanodes in microbial fuel cells for wastewater treatment (Hubenova et al., 2019). The layer-by-layer technique is another method to manipulate biofilms artificially. Rijavec et al. constructed a protective biofilm coating consisting of two strains on steel surfaces. Gramicidin S producing cells *Brevibacillus brevis* are encapsulated with artificial polyelectrolytes deposited on steel surfaces and then enclosed within a rubber elastomer layer. On the rubber elastomer layer surfaces, *Bacillus pumilus* cells were then deposited with the assistance of polyelectrolytes. They demonstrated that when applied, this artificial biofilm could affect the phylogenetic structure of the developing natural biofilm and show a possible way to reduce phylogenetic diversity and exclude undesired bacteria (Rijavec et al., 2019).

### Stage 4—Biofilm Dispersion

Biofilm dispersion is an active process that is usually initiated by native and environmental cues. By leaving bacteria to convert to their planktonic growth mode and leaving behind eroded biofilms, biofilm is believed to be more vulnerable at this stage. Therefore, it has been proposed that promoting biofilm dispersion can be one of the antimicrobial methods. However, more caution needs to be taken by using this method since it has been found the dispersed cells display different phenotypes compared to their planktonic and biofilm counterparts. Dispersing cells without efficient killing might cause a significant problem (Rumbaugh and Sauer, 2020). In addition to natural dispersion, materials can also assist this process. Iron oxide nanoparticles are one of the most studied candidates for assisting in removing established biofilms by either catalyzing free radical generation (Gao et al., 2016; Liu Y. et al., 2018; Hwang et al., 2019) or mechanical force like digging channels or mechanically erasing biofilm structure (Hwang et al., 2019; Quan et al., 2019). In addition to adding external particles, dynamic substrates showed promising results in effectively eliminating matured biofilms (Geilich et al., 2017; Wang W. et al., 2018; Gu et al., 2020). It has been studied on a catheter model. By applying a relatively high strain rate on silicon elastomer-based substrates, biofilms can be readily detached as large pieces once the applied strain reaches a critical value. Interestingly, when the applied strain rate is relatively low, biofilms can still be bound to the substrates even if the substrates are under high strains (Levering et al., 2014). Besides, by incorporating magnetic particles on top of the pillar, micron-sized pillars can be engineered to beat at a programmable frequency and force level when applying an electromagnetic field. These active surfaces effectively removed established biofilms of *uropathogenic E. coli*, *P. aeruginosa*, and *S. aureus* (Figure 4B; Gu et al., 2020). Shape memory polymers are another candidate for fabricating dynamic substrates. Gu et al. (2016b) used shape memory polymer to fabricate recessive hexagonal patterns and found a dynamic change in surface topography could remove the biofilm. However, this is a one-way shape memory polymer, which means that the material can undergo shape change only once. To overcome this drawback, Lee et al. used a reversible shape memory polymer with a shape transition temperature close to body temperature, proving that *P. aeruginosa* biofilm could be effectively removed. It was also shown that the detached biofilm cell had increased antibiotic susceptibility compared to the static control (Figure 4C; Lee et al., 2021).

## Controlling Biofilm Formation Using Synthetic Biology

The genome of bacteria determines the biofilm formation directly. Synthetic biology is a powerful technique allowing the manipulation of existing biological systems in a controlled manner (El Karoui et al., 2019; McCarty and Ledesma-Amaro, 2019). Bacteria and associated biofilms can be genetically modified to achieve their designed behaviors and functions (McCarty and Ledesma-Amaro, 2019; Mukhi et al., 2022). The following section gives examples of controlling biofilms *via* synthetic biology techniques for different applications.

### Amyloids

The biofilm secretes EPSs containing mainly polysaccharides, eDNA, and proteins. Polysaccharides constitute most biofilm mass composing glucan, fructan homopolymers, glucose/mannose/rhamnose heteropolymers, cellulose, alginate, colonic acid, and *N*-acetylglucosamine, and are highly heterogeneous across bacterial species (Nguyen, 2017). eDNA primarily relies on cellular lysis, which is an energetically inefficient system for large-scale production (Nguyen, 2017). Functional amyloids are one of the important proteins in the biofilm matrix. They comprise stacks of β-sheets aligned perpendicular to the fibril axis (Cao and Mezzenga, 2019; Gallardo et al., 2020). Unlike amyloid fibers formed upon aberrant misfolding of proteins, which are always associated with several incurable degenerative human diseases, bacterial amyloids benefit the life cycle of the biofilm. They are the key structural components of the biofilm matrix and can mediate toxicity and cell-to-cell interactions within the biofilm. Besides, amyloids are responsible for the spatial structure, morphological differentiation, surface properties, and viral protection of the biofilm (Biesecker et al., 2018; Deshmukh et al., 2018; Erskine et al., 2018; Van Gerven et al., 2018).

Curli are one of the important amyloids that have been used as building blocks for specific applications in nanotechnology due to their high stability and physical robustness (Taglialegna et al., 2016). They are non-covalent heteropolymeric filaments of CsgA and CsgB subunits where CsgA is secreted into the extracellular milieu and self-assembled into nanofibers by seeding onto a membrane-anchored nucleation protein CsgB (Van Gerven et al., 2015; Nguyen, 2017). Therefore, by fusing functional peptides or proteins into the amyloid monomers, a large-scale nanomaterial with programmable functionality can be secreted by cells (Elizabeth et al., 2016; Knowles and Mezzenga, 2016; Li et al., 2018). Nguyen et al. presented a biofilm-integrated nanofiber (BIND) system for precise genetic programming of bacterial matrix by fusing peptide domains onto the amyloid protein CsgA. Three peptides with different functions were fused to CsgA through C-terminal for extracellular self-assembly into functionalized curli nanofibers. They demonstrated the ability to engineer biofilm to template silver nanoparticles, increase adhesion to 304L stainless steel, and covalent immobilize proteins (green fluorescent protein as an example) (Nguyen et al., 2014). Similarly, mussel food protein of *Mytilus galloprovincialis* can also be fused with CsgA protein to produce adhesives with better properties like strong wet bonding strength, robustness, stability, and intrinsic fluorescence (Zhong et al., 2014). Besides, the BIND platform can also be used to immobilize enzymes for biocatalytic surface application. It has been reported that upon fusing to the SpyCatcher attachment domain, a recombinant α-amylase could be immobilized onto *E. coli* curli fibers displaying complementary SpyTag capture domains. The obtained enzymes’ immobilized biofilms were shown to be active after exposure to various adverse conditions (Botyanszki et al., 2015). Patterns are always relatively challenging to fabricate and usually require multiple fabrication steps (Shi et al., 2020). Using inducible genetic circuits and cellular communication circuits to regulate *E. coli* curli amyloid production can render amyloid fibril forming patterns either autonomously or *via* external control (Chen et al., 2014; Wang X. et al., 2018). It has been demonstrated that amyloid fibrils assembled could co-organize and synthesize inorganic nano-objects like fluorescent quantum dots (QDs) and gold nanowires, nanorods, and nanoparticles (Chen et al., 2014). Wang et al. further pushed these bio-abiotic hybrid materials to a new complexity level by engineering *E. coli* to harbor a blue-light inducible gene circuit to control the expression of CsgA through programmable light regulation; it is capable of spatiotemporally controlling nano-objects assembly. They demonstrated that discrete nano-objects and complex heterogeneous structures could be assembled hierarchically on different substrates or 3D materials of complex shapes. A minimum patterning resolution of 100 μm was achieved using light-sensing cells (Wang X. et al., 2018). Resettable pressure sensors could be fabricated using amyloid fibrils as the scaffold. With the help of inkjet printing to initiate single colony localization, engineered self-patterned bacteria could grow and subsequently facilitate the assembly of nanoparticles into a 3D dome structure. Their geometry would determine the dome structure’s response to pressure (Cao et al., 2017).

In addition to *E. coli* cells, *Bacillus subtilis* is another strain capable of this purpose. *B. subtilis’* biofilm amyloid fibers were composites of TasA and TapA as major and minor protein components, respectively. The tapA-sipW-tasA gene operon regulates the production of amyloid fibers. Huang et al. fused the extracellular amyloid-like protein Tas A with various other proteins or protein domains to endow the biofilm with new functionalities. They demonstrated this platform could make programmable materials with various functions, including intrinsic fluorescent, intact enzyme activity, and the capability of templating inorganic nanoparticles. Besides, they were also able to adjust biofilm’s viscoelastic properties to manipulate them into diverse shapes and microstructures independently or using 3D printing techniques (Huang et al., 2019). Similarly, Zhang et al. (2019) engineered amyloid protein with a mussel foot protein and hydrophobin-like protein to control adhesion.

### Biomineralization

Mineralization is a process in which biology regulates the formation of hierarchical architectural mineralized materials and is vital for nature and human activities. It is a prevalent process in which species capable of forming minerals could be found in six taxonomic kingdoms. Examples of natural mineral products include teeth, coral, protozoan shells, etc. (Yao et al., 2017; Ehrlich et al., 2021). The biomineralization process generally involves an organic matrix to form a framework, and then nucleation sites are constructed, followed by controlling crystal orientation, growth, and termination crystal growth (Sharma et al., 2021). It is generally classified into two classes—biologically induced and biologically controlled mineralization, depending on the degree of biological control exerted. The former refers to the passive interaction of environments that drive the precipitation. The latter means the organism directly controls the precipitation process, producing minerals of a specific size, morphology structure, and orientation. Some literature has already documented the detailed mechanism, which will not be covered here (Qin et al., 2020; Hoffmann et al., 2021). Depending on the strains, bacterium mediates different mineralization, including calcification, silicification, iron, Mn mineralization, and so on (Couasnon et al., 2020; Qin et al., 2020). The application of biomineralization covers many fields such as environmental science for pollutant removal (Long et al., 2021) or water treatment (Arias et al., 2019), civil engineering for artwork conservation or as construction material (Achal et al., 2015; Marvasi et al., 2020), biomedical engineering for cancer therapy, or tissue engineering (Chen Y. et al., 2019; Qin et al., 2020).

In addition, to use bacteria individually for nanoparticle fabrication, mineral generation, or pollutant degradation, there are studies treating biofilm as a type of material. Zeng et al. discovered two *Pesudoalteromonas lipolytic* variants are capable of forming wrinkled and translucent biofilms. The wrinkled biofilm was due to a nonsense mutation in *AToo_o8765*, causing overproduction of extracellular polysaccharides, while the translucent ones were due to a point mutation in *AToo_17125*. Biofilm formed by either of these two strains was proved to have antifouling activities toward larval settlement and metamorphosis of the mussel *Mytilus corucus* (Zeng et al., 2015). Liu et al. further applied the strain capable of overexpression cellulose for steel protection. This strain can effectively bind Ca^2+^ and form biomineralization organic-calcite hybrid film on the steel surface to provide protection. The film formed by living bacteria could also provide *in situ* self-healing activity (Liu T. et al., 2018). In addition to selecting natural strains, Yang et al. genetically engineered *E. coli* bacteria to express DDDEEK peptide, having a strong ability to absorb mineral ions and induce the formation of a biomineral. This engineered biofilm was shown to be capable of tolerance shear force and stayed on virtually any type of material surface. Interestingly, this biofilm-based coating not only exhibited good mineralization performance and better stability compared to the hydroxyapatite spray sample but also showed no extra immunogenicity and better osteogenicity and osseointegration after 12 weeks of implantation, demonstrating their potential applications in the biomedical field (Yang et al., 2018). Wang et al. genetically engineered *E. coli* to achieve more control over the mineralization process. CasA and mussel foot protein Mfp were fused to render higher hydroxyapatite mineralization, capacity, and interfacial binding strength. To enable *E. coli* biofilms to respond to environmental stimuli, such as light, they encode the expression of CsgA-Mfp3S-pep in the Dawn plasmid, which renders the biomass density and spatial pattern controllable. Interestingly, bacteria cells inside the composite remained alive and capable of responding to environmental stimuli after mineralization (Wang Y. et al., 2021).

### Other Applications

Bacterial metabolic activities are important when they are applied for fermentation or remediation. Bacteria in their biofilm form have been reported to increase the production of value-added products and secondary metabolites. Biofilm reactors, where biofilms were immobilized onto surfaces, have been widely accepted as a promising technology in wastewater treatment, pollutant removal, and production of alcohol, organic acid, enzymes, syngas, and many other value-added products (Mahdinia et al., 2019; Abdelfattah et al., 2020; Gunes, 2021). Biofilm’s properties such as adhesion strength, biomass, and population are closely related to its efficiency. Synthetic biology techniques can be applied to genetically modify strains to significantly improve their performance. For example, the poor adhesion capacity of stain *B. Subtilis* 168 was the main issue that limited their application in biofilm reactors. After insertion of the *sfp* and *epsC* genes together with the deletion of the *sepF* gene, the biofilm’s form by this mutant strain showed increased adhesion and surfactin production (Brück et al., 2019). *Pseudomonas putida* is a widely used strain in bioremediation due to its remarkable ability to degrade pollutants; however, most of the laboratory-adapted *P. putida* strains suffer from weak biofilm formation abilities. Benedetti et al. genetically programmed their intracellular c-di-GMP levels using an inducible and tightly controlled genetic device for heterologous expression of diguanylate cyclase and phosphodiesterase genes. They then proved this biofilm formed by engineered strain displayed high dehalogenase activity (Benedetti et al., 2016). In addition to monostrain biofilms, microbial consortia behaviors could also be controlled. By manipulating communication networks, regulating gene expressions, and engineering syntrophic interactions, it is possible to control individual species’ population, distribution, and spatial organization in a microbial consortium (McCarty and Ledesma-Amaro, 2019).

## Conclusion and Future Perspectives

Bacteria tend to form biofilm as their preferred life form compared to planktonic ones once attached to a surface. Hence, manipulating the substratum properties and bacteria’s genome can both influence bacterial behavior and biofilm formation. Substrate’s properties have effects on four stages of biofilm formation. For example, surface topography, stiffness, wettability, and zeta potential can affect bacteria adhesion, such as the amount and location of the attached bacteria, the direction of movement, and viability. These factors, in turn, affect bacteria proliferation, biofilm morphology, antibiotic resistance, and biofilm dispersion. On the other hand, synthetic biology techniques are powerful tools capable of genetically modifying bacteria. These techniques directly change bacteria’s functions and metabolic activities and then generate the designed biofilms. Amyloids and biomineralization are two typical biofilms that can be designed and used as materials.

This review discussed the possibilities of controls and uses of biofilms from materiobiology and synthetic biology perspectives. To date, these two perspectives are rarely combined to produce unique biofilms. The main reason is that the two areas claim different standpoints, i.e., materials researchers usually see biofilms as toxins while synthetic biologists see these as biomaterials. In addition, while a vast amount of literature in materials science tries to avoid biofilm formation, the understanding of manipulating biofilms using the materiobiology approach is still insufficient, especially in the mechanism part. Besides, many of these studies only examined the biofilm’s adhesion stage. Consequently, controversial conclusions were obtained, such as the effect of stiffness and roughness on biofilm formation. Thus, further studies on the bacteria-surface interactions and long-term studies are necessary to gain better control properties of biofilm. On the other hand, designing biofilms using the synthetic biology approach is insufficient. We propose that combining these two techniques in the future could give better control over biofilm formation and properties.

## Author Contributions

P-YW conceived the manuscript. YS and P-YW designed the structure of the review. YS and TC collected the literature. YS, TC, and P-YW wrote the manuscript. YS, PS, and P-YW proofread the manuscript. All authors approved the final version of the manuscript.

## Conflict of Interest

The authors declare that the research was conducted in the absence of any commercial or financial relationships that could be construed as a potential conflict of interest.

## Publisher’s Note

All claims expressed in this article are solely those of the authors and do not necessarily represent those of their affiliated organizations, or those of the publisher, the editors and the reviewers. Any product that may be evaluated in this article, or claim that may be made by its manufacturer, is not guaranteed or endorsed by the publisher.
